# Early-life heat stress exposure impacts dairy calf feeding and thermoregulatory behavior

**DOI:** 10.3168/jdsc.2021-0110

**Published:** 2021-11-25

**Authors:** Bethany Dado-Senn, Katie N. Gingerich, Kelsey C. Horvath, Sena L. Field, Marcela G. Marrero, Fiona Maunsell, Emily K. Miller-Cushon, Jimena Laporta

**Affiliations:** 1 Department of Animal and Dairy Sciences, University of Wisconsin-Madison, Madison 53706; 2 Department of Animal Sciences, University of Florida, Gainesville 32608; 3 Department of Large Animal Clinical Sciences, School of Veterinary Medicine, University of Florida, Gainesville 32610

## Abstract

Heat stress has well-known influences on dairy calf physiology, but less is understood about calf behavioral responses to heat stress. Herein, we evaluated milk replacer intake, standing activity, and lying behaviors of calves exposed to prenatal or postnatal heat stress or both. Holstein calves were born to dams experiencing heat stress (HT; shade of a freestall barn) or cooling (CL; shade, fans, and soakers) during late gestation [~44 d before calving, prenatal; mean daily temperature-humidity index (THI) = 78]. They were then subsequently exposed to postnatal heat stress (shade and natural ventilation of an open-sided barn) or cooling (shade of the barn and forced ventilation by fans) from birth to weaning (56 d; mean daily THI = 77; n = 12 per prenatal × postnatal treatment). Heat stress was confirmed by elevated respiration rate and rectal temperature of the prenatal dam and the postnatal calf. Calves were group-housed with automatic milk feeders, from which milk replacer (MR) intake was assessed. Calf behavior was monitored using loggers and video. Postnatal-HT calves tended to consume less MR per hour in the late morning and drank less MR per visit relative to postnatal-CL calves. Postnatal-HT calves spent more time lying laterally and less time lying sternally in a tucked position during overnight hours. Prenatal-HT calves stood longer across the day, particularly overnight, compared with prenatal-CL calves. This study characterized behavioral responses of preweaning dairy calves exposed to chronic heat stress or active cooling during early-life developmental windows.

## Highlights

• In utero–heat-stressed calves stand longer relative to in utero–cooled counterparts.
• Postnatal heat stress lowers calf milk replacer intake, particularly in the late morning.
• Calves under postnatal heat stress alter thermoregulatory lying postures overnight.

## Graphical Abstract

**Figure:**
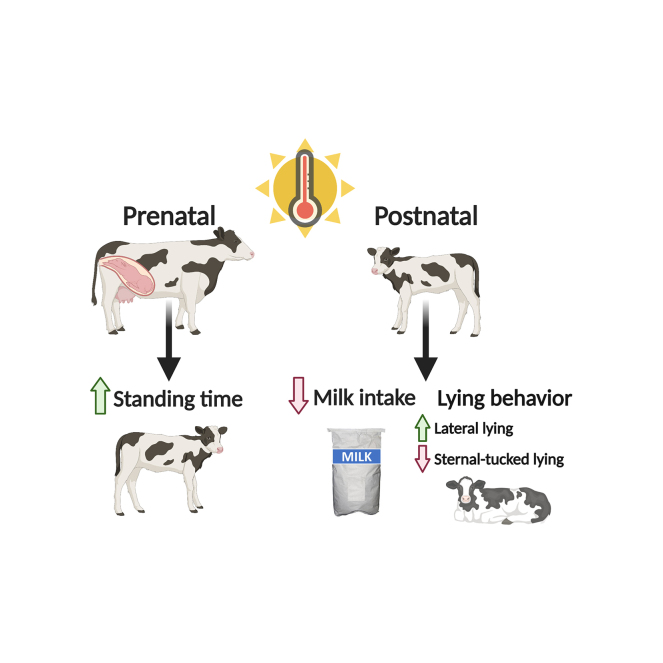
**Summary:** Exposure to prenatal heat stress increases calf standing time in the preweaning period, whereas direct exposure to postnatal heat stress preweaning leads to decreased milk replacer intake and altered lying behaviors in dairy calves.

Rising global temperatures impair dairy cattle welfare, productivity, and profitability (Polsky and von Keyserlingk, 2017; Becker et al., 2020). Physiological heat stress occurs when increased ambient temperature pushes homeotherms, such as dairy cattle, past the upper critical temperature limit of their thermoneutral zone. Homeotherms acclimate to maintain thermoneutrality by adjusting behavior and physiology to reduce heat production and increase heat loss (Bianca, 1968; Yousef, 1987; Van Os, 2019). Behavioral adaptations are the first response, as these can be executed quickly and usually require less energy (Hillman, 2009). Notable mature cow thermoregulatory behaviors include altered volume and patterns of feed and water intake; seeking active air movement and shade and water sources; increased standing time; and reduced activity (Hillman, 2009; Polsky and von Keyserlingk, 2017; Van Os, 2019). These adaptations lower core body temperature or reduce the rate of heat accumulation in cattle (West, 2003; Gebremedhin et al., 2011; Allen et al., 2015).

Exposure to elevated ambient temperatures during either prenatal or postnatal developmental windows can also lead to negative consequences in dairy calves. Heifers exposed to heat stress in late gestation weigh less at birth and weaning, have compromised immunity, and produce less milk upon entering the milking herd (Monteiro et al., 2016; Laporta et al., 2017; Tao et al., 2019). Postnatal heat stress in preweaning dairy calves triggers physiological responses such as elevated respiration rates (**RR**; Carter et al., 2014), decreased nutrient intake (Broucek et al., 2008), and reduced ADG (Hill et al., 2011; López et al., 2018). Still, very little is known about the impact of heat stress in these periods on calf thermoregulatory behaviors and feed intake patterns. Limited literature suggests that calves exposed to heat stress in late gestation spend less time standing relative to calves born to cooled dams (Laporta et al., 2017). Postnatal studies in dairy and beef calves showed an increase in standing time as environmental temperature-humidity index (**THI**) increases (Carter et al., 2014; Kamal et al., 2016). A better understanding of the thermoregulatory behaviors of heat-stressed dairy calves is needed to develop management interventions to aid in identifying and developing effective heat abatement methods for youngstock.

The objective of the current study was to characterize milk replacer (**MR**) intake and standing and lying behavioral patterns of dairy calves exposed to heat stress or cooling during late gestation and preweaning early-life developmental stages. We hypothesized that calves exposed to postnatal heat stress would reduce overall feed intake, meal size, and feeding visits; stand longer; and display lying postural behaviors to promote thermoregulation for longer (i.e., lying in a lateral position and pen-mate avoidance) relative to calves offered postnatal heat stress abatement. We also hypothesized that the prenatal intrauterine environment would influence feed intake and standing time, especially when followed by postnatal heat stress.

A detailed description of the experimental design can be found in Dado-Senn et al. (2020), which reported calf physiological outcomes to pre- and postnatal heat stress. In summer 2018 at the University of Florida Dairy Unit, multiparous pregnant Holstein cows were randomly enrolled into a dry period treatment of either heat stress (**HT**, shade of the freestall barn) or cooling (**CL**, shade plus fans and water soakers) in sand-bedded freestall pens where they remained until calving (i.e., late gestation; 44 ± 5 d). Forty-eight bull and heifer calves born to these dams were used in this study and experienced prenatal heat stress (**pre-HT**, n = 24) or cooling (**pre-CL**, n = 24) through the intrauterine environment. Postnatal treatments were initiated at 2 d of age between June and September. To assess environmental heat stress, ambient temperature and relative humidity data were recorded every 15 min and averaged per hour using Hobo Pro Series Temp Probes to calculate THI average from the National Research Council equation (NRC, 1971). Calves were assigned to group automatic feeder pens (n = 8 pens) with access to the shade of an open-sided, sand-bedded barn (**post-HT**, n = 24) or the shade plus 2 fans (**post-CL**, n = 24) until weaning was completed (56 d of age). Thus, the 2 × 2 factorial design resulted in 4 treatments (n = 12 calves per treatment): (1) cooled prenatally and postnatally (**CL-CL**), (2) cooled prenatally and heat-stressed postnatally (**CL-HT**), (3) heat-stressed prenatally and cooled postnatally (**HT-CL**), and (4) heat-stressed prenatally and postnatally (**HT-HT**). Postnatal-HT pens housed n = 3 CL-HT and n = 3 HT-HT calves, while postnatal-CL pens had n = 3 CL-CL and n = 3 HT-CL calves. Due to the 2 × 2 factorial study design and distribution of both prenatal treatments to one postnatal treatment pen, the individual calf was considered the experimental unit. The sex distribution per treatment was as follows: CL-CL = 7 bulls, 5 heifers; CL-HT = 8 bulls, 4 heifers; HT-CL = 6 bulls, 6 heifers; HT-HT = 5 bulls, 7 heifers. No animals were excluded in the experiment. Data were analyzed from wk 3 to 8 of age (postnatal) to allow for pen acclimation and feeding training. The University of Florida Institutional Animal Care and Use Committee approved all treatments, and experiments were conducted in accordance with their rules and regulations.

Calves were allotted up to 10 L/d of MR (UF Special 28/15 Bova DFB Medicated, Southeast Milk; mixing rate 150 g/L) in maximum 3-L increments via automatic feeder (DeLaval CF1000X). Calves were trained thrice daily to consume MR until they accessed the feeder without assistance. Milk weaning began at 42 d of age as a 2-L reduction every 2 d from 10 to 0 L and ended at 52 d of age. Up to 3 kg/d starter grain concentrate was provided via automatic feeder, and water was provided ad libitum. The automatic feeder recorded daily MR intake, number of feeder visits both rewarded and unrewarded (i.e., milk allowed vs. not allowed), and MR intake per visit, which were averaged weekly for analysis. Milk replacer intake per hour was also calculated from 4 distinct periods: (1) 0000–0700 h (early a.m.), (2) 0700–1300 h (late a.m.), (3) 1300–1900 h (early p.m.), and (4) 1900–2400 h (late p.m.) from 3 to 6 wk to assess intake patterns in each period. Intake, speed, and visits were assessed from 3 to 8 wk of age.

Calf standing behavior was monitored daily from 3 to 8 wk of age using electronic data loggers (Hobo Pendant G Data Logger; Onset Computer Corp.) attached to the calf's rear leg (left inner leg or right outer leg; validated in calves by Bonk et al., 2013). Loggers were removed and replaced weekly. Data were captured from loggers and transformed from planar to time and bout data for statistical assessment (Ledgerwood et al., 2010; Chapinal et al., 2011). Standing activity was recorded as total daily standing time (min/d), daily standing bout frequency (bouts/d), daily standing bout duration (min/bout), total hourly standing time (min/h) from 3 to 8 wk of age, and hourly standing time (min/h) at 4, 6, and 8 wk of age separately. These weeks were selected to account for potential changes as the calf aged, as well as specific management events: disbudding (wk 4), initiation of weaning (wk 6), and completion of weaning (wk 8). Calves were handled minimally throughout the day, apart from environmental and thermoregulatory measurements at 0700, 1300, and 1900 h, which are described in detail in Dado-Senn et al. (2020).

Calf lying behavior was measured in a subset of calves (n = 6 to 7 per treatment) from video recorded by cameras (high-definition day/night cameras; AXIS M2026-LE camera, Axis Communications Inc.) mounted above each pen. Behavioral data were collected from calves for 3 consecutive days (22.8 ± 2.5 d of age). For each pen, the behaviors of animals (identified by unique coat markings) were recorded continuously for 24 h. Behavior start and end times were recorded using Behavioral Observation Research Interactive Software (BORIS; 1/100 s accuracy; Friard and Gamba, 2016). Observed behaviors include calf lying posture (i.e., lateral, sternal, or sternal-tucked), social lying (i.e., social or distant), and calf lying location within the pen (i.e., inside, center, or outside). Two observers were used to characterize these behaviors from video. Inter-observer reliability was calculated, with Cohen's kappa in BORIS exceeding 0.90 for observer comparisons.

All data except automatic feeder visits were analyzed as a 2 × 2 factorial design using the MIXED procedure in SAS version 9.4 (SAS Institute). Automatic feeder visits were analyzed by generalized linear mixed models using PROC GLIMMIX with a Poisson distribution and log function. Main effects for both MIXED and GLIMMIX models included prenatal treatment, postnatal treatment, time (hour, day, or week as repeated measure), and all interactions. Time point comparisons were assessed by multiple comparisons. Milk replacer intake per hour at each period (i.e., early/late a.m. and early/late p.m.) was assessed separately. The random effects of pen (nested within treatment), calf ID (nested within treatment × pen), and time × pen (nested within treatment) were included following recommendations from St-Pierre (2007) to account for postnatal pen effects. Sex and THI at time of treatment were included as covariates, and the first-order autoregressive covariance structure (AR-1) or compound symmetry (CS) was used as the covariate structure. Residuals were tested for normality and data were log-transformed if needed (i.e., daily standing duration and center lying behavior). These data were back-transformed into the original scale for visualization. Significance was declared at *P* ≤ 0.05 and tendency was declared at 0.05 < *P* ≤ 0.10. Data were reported as least squares means (LSM) ± standard errors (SE) of the prenatal treatment × time, postnatal treatment × time, or prenatal treatment × postnatal treatment × time, depending on the significant effect(s).

Physiological thermoregulatory methods and outcomes are described in Dado-Senn et al. (2020). Briefly, pre-HT and pre-CL were achieved as indicated by continuously high THI (daily mean = 77.9 ± 1.7) in treatment pens and reduced vaginal temperatures (38.9 vs. 39.1 ± 0.1°C; *P* < 0.01) and RR (54.6 vs. 65.3 ± 1.2 bpm; *P* < 0.01) in the CL relative to HT dams. Mean postnatal daily THI was 77, with the lowest THI measures recorded in the early a.m. and late p.m. and elevated THI measures in the late a.m. and early p.m. periods (Dado-Senn et al., 2020). The post-CL pens, equipped with shade and fans, had increased air speed (2.05 vs. 0.15 ± 0.02 m/s, *P* < 0.01) that lowered calf thermoregulatory responses, including RR (47.9 vs. 56.2 ± 0.8 bpm; *P* < 0.01), rectal temperature (39.0 vs. 39.1 ± 0.02°C; *P* < 0.01), and hourly rectum surface temperature compared with post-HT calves with access to shade but no fans (Dado-Senn et al., 2020).

Postnatally heat-stressed calves tended to consume less MR during the late a.m. hours relative to post-CL calves, driven by reduced intake in HT-HT calves (*P* = 0.09; Figure 1A). Overall, post-HT calves consumed less MR per day and per hour than post-CL calves (7.5 vs. 8.3 ± 0.3 L/d, *P* = 0.02; 0.32 vs. 0.35 ± 0.1 L/h, *P* = 0.02; Figure 1A). Prenatal treatment did not affect calf feeding patterns throughout the day (*P* > 0.22). Although calf total daily visits to the automatic feeder were not affected by postnatal treatment (Figure 1B), post-HT calves had fewer unrewarded visits (*P* = 0.05; Figure 1C) and more rewarded visits, particularly at 5 to 8 wk of age (*P* < 0.01; Figure 1D) compared with post-CL calves. This discrepancy in visits is likely attributed to post-HT calves consuming less MR per visit (*P* = 0.01; Figure 1E), as the automatic feeder allotted a maximum intake over specific periods.

**Figure 1:**
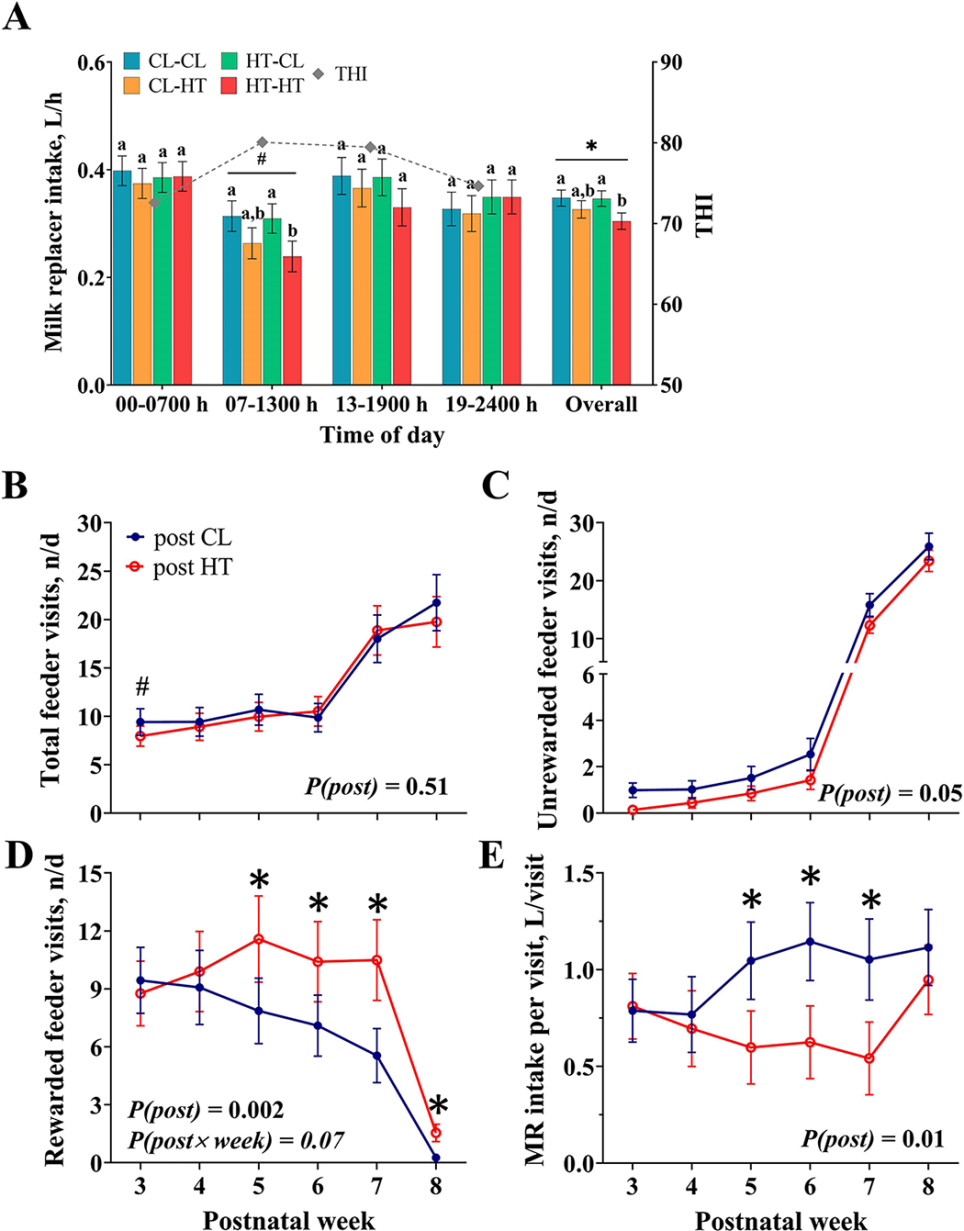
Feeding behavior of dairy calves exposed to prenatal (pre, ~46 d) and postnatal (post, 56 d) cooling (CL) or heat stress (HT): CL-CL, CL-HT, HT-CL, HT-HT, n = 12 per treatment). Milk replacer (MR) intake per hour from 3 to 6 wk of age for specific periods of the day: 0000–0700 h (early a.m.), 0700–1300 (late a.m.), 1300–1900 h (early p.m.), and 1900–2400 h (late p.m.) (A). Average hourly mean temperature-humidity index (THI) for each period is indicated by solid gray lines on the secondary y-axis (A). Number of calf visits to the MR auto feeder per day from 3 to 8 wk of age including total (B), unrewarded (C), and rewarded visits (D), as well as the amount of MR consumed per rewarded visit (E). Data are graphed using the LSM ± SE of the prenatal × postnatal interaction (A) or postnatal effect over time (B–E; n = 24 per treatment). * (*P* ≤ 0.05) and # (0.10 ≥ *P* > 0.05) indicate significance and tendency, respectively, for postnatal treatment. Different letters (a, b) indicate significant differences (*P* ≤ 0.05) between individual treatments within a specific time period.

There was a postnatal treatment × week interaction for daily standing time, whereby post-HT calves stood for less time per day at 3 wk of age but increased standing time per day at 8 wk of age (*P* ≤ 0.02; Figure 2A) compared with post-CL calves. Postnatal HT calves also had an increase in standing bouts per day relative to post-CL calves (20.4 vs. 18.9 ± 0.6 bouts/d; *P* = 0.05). There was a postnatal treatment × hour interaction (*P* < 0.001; Figure 2B) for hourly standing time as post-CL calves stood longer in some a.m. and early p.m. hours whereas post-HT calves stood longer at 1500, 1900, and 2000 h. There were also prenatal main effects for standing time, whereby pre-HT calves had greater daily standing time relative to pre-CL calves (462.8 vs. 433.4 ± 9.9 min/d; *P* = 0.02; Figure 2C), possibly driven by a tendency for longer standing bouts (25.8 vs. 24.0 ± 0.9 min/bout; *P* = 0.09). Further, there were prenatal treatment and treatment × hour interactions for hourly standing time, as pre-HT calves stood longer relative to pre-CL calves, particularly in early a.m. and late p.m. hours from 3 to 8 wk of age and at 4, 6, and 8 wk of age separately (*P* ≤ 0.03; Figure 2D-G).

**Figure 2:**
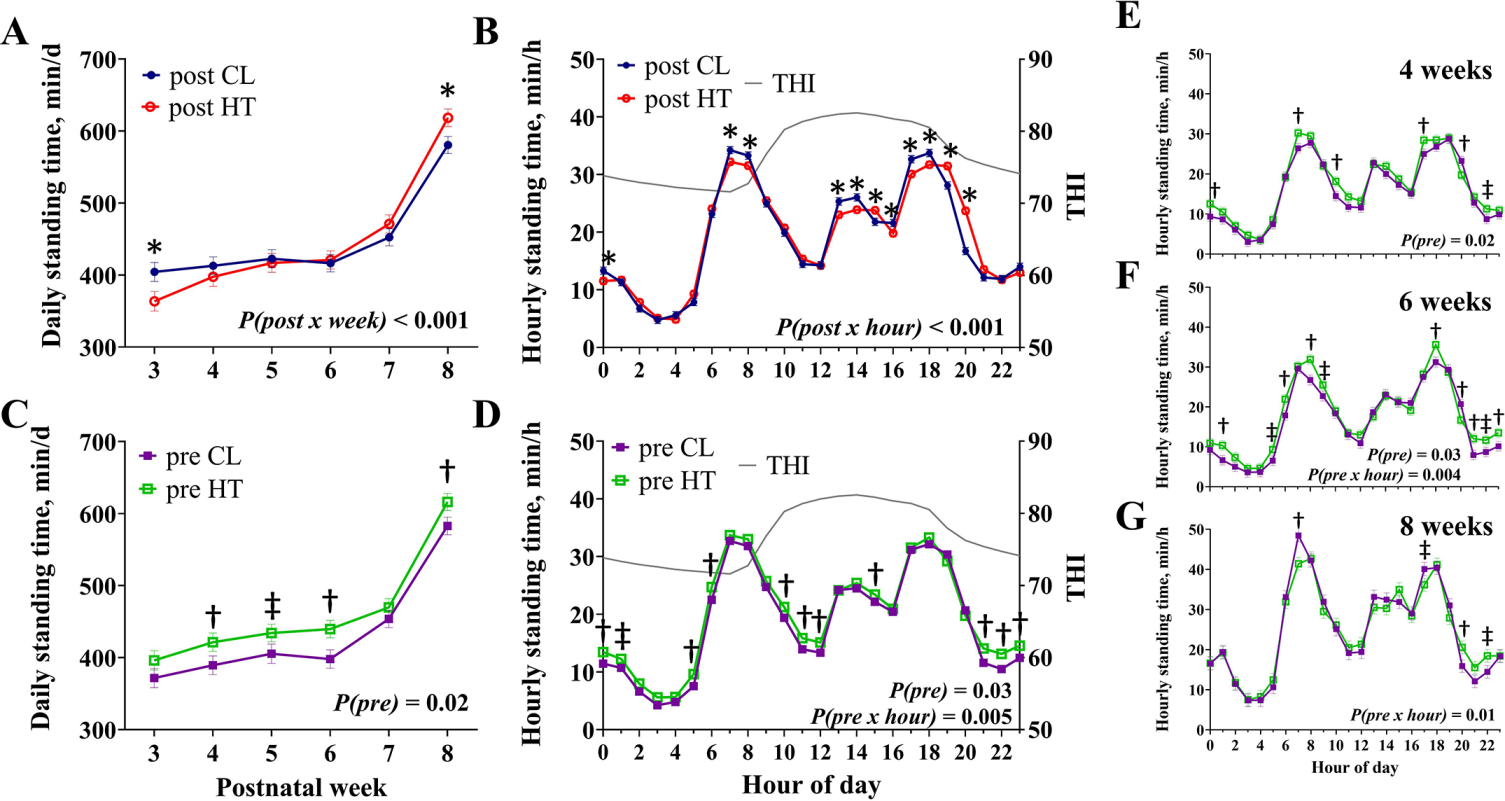
Calf daily total standing time and total hourly standing time for postnatal effect (post; A, B) and prenatal effect (pre; C, D), plus hourly standing time at 4, 6, and 8 wk of age (E–G). Dairy calves were exposed to prenatal (~46 d) and postnatal (56 d) cooling (CL) or heat stress (HT). Data are graphed using the LSM ± SE of the postnatal (A, B) or prenatal effect over time (C–G, n = 24 per treatment). Hourly mean temperature-humidity index (THI) is indicated by solid gray lines on the secondary y-axis (B, D). * (*P* ≤ 0.05) indicates significance for postnatal treatment; † (*P* ≤ 0.05) and ‡ (0.10 ≥ *P* > 0.05) indicate significance and tendency, respectively, for prenatal treatment.

Calf postures, including lateral and sternal-tucked, and center lying were influenced by heat stress exposure (Figure 3). In the early a.m. and late p.m. hours, pre-CL calves spent more time lying laterally relative to pre-HT calves, whereas post-HT calves spent more time lying laterally relative to post-CL calves (*P* ≤ 0.02; Figure 3C). Thus, CL-HT calves were lateral for more time overnight relative to other treatment groups (*P* ≤ 0.05). Similarly, post-HT calves spent less time in the sternal-tucked position relative to post-CL calves, particularly in the early a.m. and late p.m. hours (*P* ≤ 0.05; Figure 3D). There was a postnatal effect for center lying as post-CL calves spent more time lying in the center of the pen, where airspeed was maximized (3.44 vs. 0.15 ± 0.05 m/s, *P* < 0.01; Figure 3A), relative to post-HT calves (7.6 vs. 4.0 ± 1.9 min/h, *P* = 0.03; Figure 3E). There were no treatment effects for other lying behaviors (*P* > 0.16).

**Figure 3:**
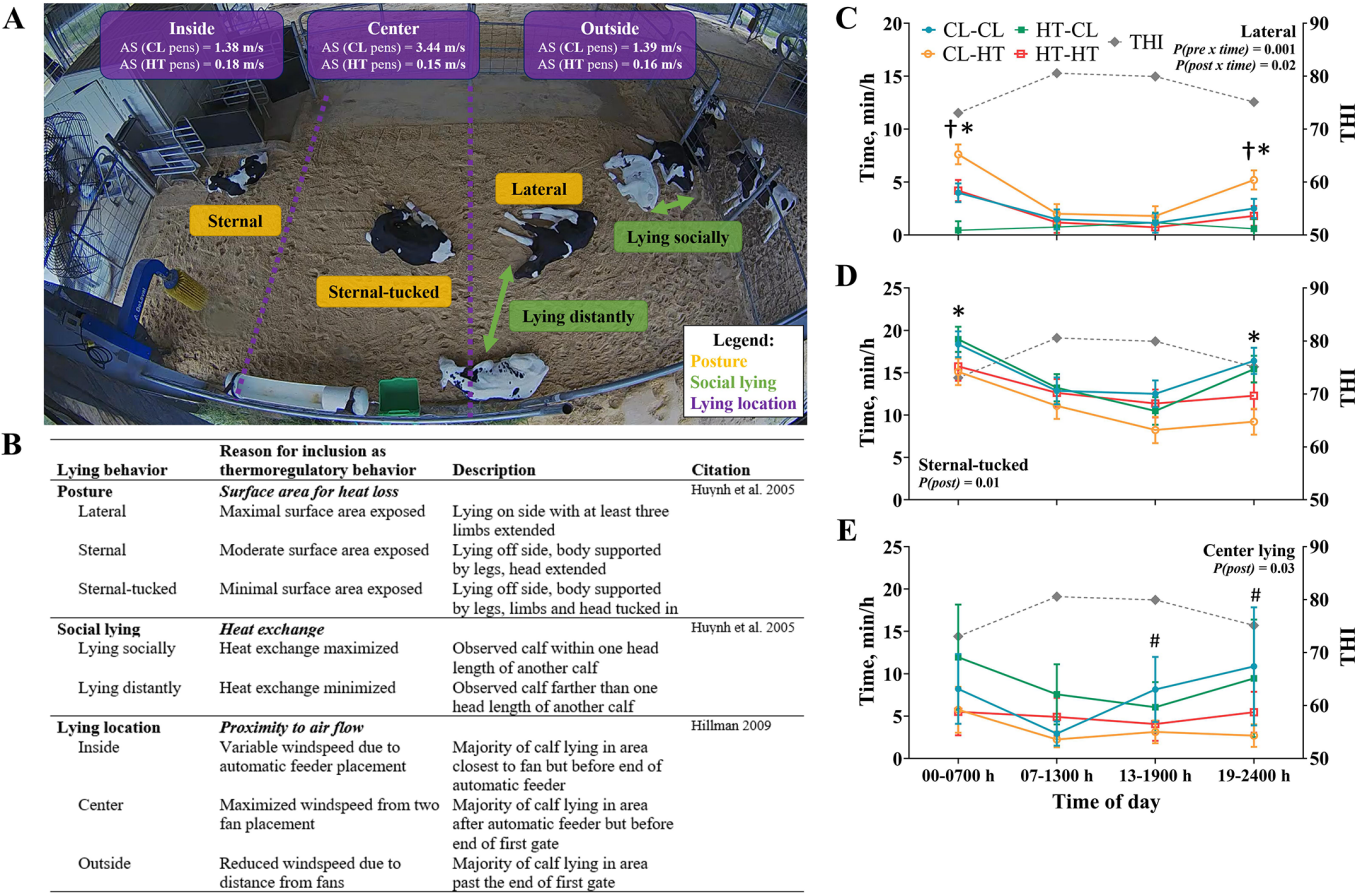
Visual (A) and descriptive (B) representation of calf daily thermoregulatory lying behaviors at 3 wk of age as observed through video monitoring and BORIS software. Dairy calves were exposed to prenatal (pre, ~46 d) and postnatal (post, 56 d) cooling or heat stress (CL-CL, CL-HT, HT-CL, HT-HT, n = 6 to 7 per treatment). Depicted is a post-CL pen (n = 3 CL-CL calves, n = 3 HT-CL calves) with 2 fans. Corresponding air speeds (AS) for each pen location are described in the purple boxes. Postnatal-HT pens are mirrored except for fans. Lying behaviors including lateral posture (C), sternal-tucked posture (D), and center lying (E) throughout the day. Data are graphed using the LSM ± SE of the prenatal × postnatal interaction over time. Specific periods of day: 0000–0700 h (early a.m.), 0700–1300 (late a.m.), 1300–1900 h (early p.m.), and 1900–2400 h (late p.m.). Average hourly mean temperature-humidity index (THI) for each period is indicated with solid gray lines on the secondary y-axis (C-E). † and * (both *P* ≤ 0.05) indicate significance for prenatal or postnatal treatment, respectively; # (0.10 ≥ *P* > 0.05) indicates tendency for postnatal treatment.

Herein, we describe the behaviors of group-housed dairy calves exposed to chronic heat stress or active cooling across early-life developmental windows. Dairy calves under postnatal heat stress had reduced MR intake, particularly during late a.m. hours, and consumed less MR per automatic feeder visit. The decline in voluntary feed intake under hyperthermic conditions is a well-known behavioral indicator of heat stress, because acclimation to heat stress promotes reduction of energy intake and heat generation (Brobeck, 1948). There is an inverse relationship between elevated ambient temperature and feed intake in lactating dairy cows, partially due to high metabolic heat loads (West, 2003; Spiers et al., 2004; Bernabucci et al., 2010), but the effect of heat stress on preweaning calf feed intake is sparsely reported. Calves born in hot summer months are reported to have reduced calf starter grain intakes and BW gain compared with those born in cooler winter months, but MR intake was not recorded in those studies (Broucek et al., 2008; López et al., 2018). Herein we found that automatic feeder MR consumption was reduced in postnatally heat-stressed calves, especially those continuously heat-stressed (i.e., HT-HT), in the late morning hours. Despite similarly elevated THI, this difference did not carry into the early evening hours, possibly due to the stimuli of surrounding activity at this time (i.e., feeding of individually housed calves at 1530 h) that could negate any treatment effects (Miller-Cushon and DeVries, 2015). Together, our results have management implications for dairy producers, as provision of heat stress abatement may maximize MR intake for calves in a group-housing, automatic-feeder system. Maintaining or improving MR intake through strategic management could benefit calf welfare and both immediate and long-term productive responses (Silanikove, 2000; Soberon et al., 2012; Orellana Rivas et al., 2020).

Another commonly measured thermoregulatory behavior in dairy cattle is standing and lying behavior. It is thought that dairy cows respond to heat stress by increasing standing time to expose more surface area for evaporative and sensible heat loss with implications for comfort and performance (Haley et al., 2000; Cook et al., 2007; Allen et al., 2015). Limited studies in dairy and beef calves further describe increased standing time or lying bout frequency under heat stress conditions, indicating increased discomfort (Carter et al., 2014; Kamal et al., 2016; Kovács et al., 2018). In the present study, it is difficult to determine an effect of direct postnatal heat stress exposure on calf standing time, because there were periods of increased standing time in both postnatal CL and HT calves across days and hours of the day. The variability in postnatal CL and HT standing activity may be attributed to changes in calf development and feeding activity.

Prenatal heat stress exposure has been shown to affect neonate postnatal activity (Shiota and Kayamura, 1989; Vom Saal et al., 1991). Herein, prenatally heat-stressed calves had longer daily standing times and stood longer for most hours of the day, particularly during late p.m. and early a.m. hours. It has been hypothesized that prenatal stressors lead to long-term consequences on postnatal organ structure and function, leaving the offspring susceptible to disease and physiological dysfunction (Reynolds et al., 2019). In line with this theory, we hypothesize that prenatal heat stress predisposes calves to greater thermal sensitivity and altered thermoregulation postnatally, as evidenced by differences in thermoregulatory organ structures (i.e., skin and sweat glands) upon in utero heat stress (Davidson et al., 2021) and increased heat loads. For instance, the calves in this study that were continuously exposed to both pre- and postnatal heat stress were shown to have elevated rectal temperatures under high THI (i.e., 1300 h, Dado-Senn et al., 2020). The increased standing of prenatally heat-stressed calves herein might be an attempt to optimize heat loss overnight after internal heat accumulation when THI is lower (West, 2003). However, previous research from our group found that prenatal heat-stressed calves had reduced standing time compared with prenatally cooled calves, but calves were only monitored at 1, 7, and 8 wk of age and were individually housed (Laporta et al., 2017). Additionally, factors such as health status or postnatal ambient temperature, as postnatal measures were collected from July to December, may have influenced outcomes but were not recorded in the above-mentioned study.

Although lying down inhibits maximal surface area exposure, certain lying behaviors such as cool-seeking (i.e., lying near sources of shade, water, or air movement), lying posture, and pen-mate avoidance can promote heat loss (Huynh et al., 2005; Hillman, 2009; Van Os, 2019). Research in porcine models has found elevated lateral lying and decreased social lying in pigs under high ambient temperatures (Aarnink et al., 2001; Huynh et al., 2005). Similarly, the postnatally heat-stressed calves in our study spent more time lying laterally and less time lying sternal-tucked in the late p.m. and early a.m., which could maximize surface area for heat loss overnight. Further, calves provided active cooling postnatally (i.e., post-CL) spent more time lying in the center of the pen under maximized air speeds relative to calves not provided active cooling, which supports the idea of cool-seeking postnatally. However, responses varied greatly between calves. Other factors influencing lying behaviors such as behavior of other calves in the pen, individual-specific social lying preferences, and the possibility of persistent individual rest location preferences may have contributed to outcomes and variability; thus, behaviors should be interpreted with caution (Færevik et al., 2007).

In summary, both prenatal and postnatal heat stress triggered specific aspects of preweaning dairy calf thermoregulatory behavior. Direct postnatal heat stress exposure decreased MR intake, particularly in the late morning, and prompted increased time in thermoregulatory lying postures overnight. In contrast, prenatally heat-stressed calves stood longer, especially overnight. This work will allow for more immediate recognition of thermal discomfort and intervention of heat stress abatement strategies, promoting dairy calf health, welfare, and short- and long-term productivity.
